# Living Donors’ Age Modifies the Impact of Pre-Donation Estimated Glomerular Filtration Rate on Graft Survival

**DOI:** 10.3390/jcm12216777

**Published:** 2023-10-26

**Authors:** Manuela Almeida, Catarina Ribeiro, José Silvano, Sofia Pedroso, Sandra Tafulo, La Salete Martins, Miguel Ramos, Jorge Malheiro

**Affiliations:** 1 Department of Nephrology, Centro Hospitalar Universitário de Santo António (CHUdSA), 4099-001 Porto, Portugal; u13204@chporto.min-saude.pt (C.R.); u14414@chporto.min-saude.pt (J.S.); sofiapedroso.nefrologia@chporto.min-saude.pt (S.P.); lasaletemartins.nefrologia@chporto.min-saude.pt (L.S.M.); jmalheiro.nefrologia@chporto.min-saude.pt (J.M.); 2 UMIB—Unit for Multidisciplinary Research in Biomedicine, ICBAS—School of Medicine and Biomedical Sciences, University of Porto, 4050-348 Porto, Portugal; sandra.tafulo@ipst.min-saude.pt; 3 Instituto Português do Sangue e da Transplantação, 4200-139 Porto, Portugal; 4 Department of Urology, Centro Hospitalar Universitário de Santo António (CHUdSA), 4099-001 Porto, Portugal; miguelsilvaramos@gmail.com

**Keywords:** age, graft survival, living donor, glomerular filtration rate, kidney transplantation, medically complex living donors

## Abstract

Background: The global scarcity of organs for kidney transplants (KTs) has led to the increased acceptance of living donors (LDs) with minor abnormalities to increase the donor pool.. We sought to evaluate the effects of some of these LDs’ clinical characteristics (older age, borderline renal function, hypertension, dyslipidemia, smoking, and obesity) on graft outcomes. Methods: We studied 352 recipients of LDKTs (1998–2020). Firstly, considering the recipients and KT variables, we identified relevant predictors of overall and censored graft failure (GF). Then, adjusting for these predictors, we explored LD variables as predictors of overall and censored GF in a multivariable Cox model. Results: The recipients from LD with higher eGFR (≥90 mL/min/1.73 m^2^) had significantly better overall and censored graft survival GS) at 15 y after KT (respectively, 67 and 75% vs. 46 and 46%, *p* < 0.001). Importantly, none of the remaining LD factors which were evaluated (hypertension, dyslipidemia, smoking, proteinuria, and obesity) were independent predictors of GF. In recipients from LDs < 50 y, having an eGFR < 90 was an independent predictor of overall GF [adjusted HR (95%CI) of 2.578 (1.120–5.795)] and censored GF [adjusted HR (95%CI) of 3.216 (1.300–7.959)], compared to recipients from LDs with eGFR ≥ 90. Contrarily, when donors were older, no difference in the risk of GF was observed between eGFR categories. Conclusion: In our cohort, lower pre-donation eGFR had an impact on GS only in younger LDs. An age-adjusted eGFR cutoff may be pursued for improved donor admissibility.

## 1. Introduction

Chronic kidney disease (CKD) is a global health problem affecting more than 10% of the general population worldwide, amounting to >800 million individuals [1]. CKD progresses to end-stage renal disease (ESRD) in approximately 2% of patients overall [1,2]. Kidney transplantation (KT) is the best treatment for ESRD. It is associated with improved survival and quality of life [2,3]. After KT, an increase in overall lifespan of 10 years is expected, from 3 years in older recipients to 17 years in the younger ones who would benefit the most from KT [3]. Unfortunately, severe organ shortage for kidney transplants is a worldwide problem [1,4]. Concerted efforts have been taken to increase the donor pool. However, the supply of donors (both living and deceased) is far lower than the need, resulting in an enormous number of qualified patients remaining on the wait-list and thousands being removed from the list every year because of death or becoming too sick for transplantation [2,4]. Portugal has a median wait-list time of around 5 years, and the mortality rate in wait-listed individuals is more than 5% each year [5]. Although this country has one of the highest deceased donor rates of kidney transplant worldwide, at 38.5 pmp in 2021, the rate of living donor is not impressive, at 3.5 pmp the same year [6].

A living donor (LD) kidney transplant (KT) is the preferred treatment for end-stage renal disease (ESRD) [7]. Living donor (LD) KTs increase organ availability, decrease time on the waiting list, allow for pre-emptive transplantation, and improve graft and patient survival [7,8]. Not all LD organs have the same quality, and donor specificities may affect recipient outcomes [9].

Increasingly, the shortage of organs leads to organ acceptance from LDs with borderline medical or surgical abnormalities, potentially associated with worse graft and recipient outcomes [7,9]. These donors are usually referred to as medically complex living donors (MCLDs), such as the elderly or those with obesity, vascular complexity, or a family history of ESRD [10].

The practice patterns of acceptance of MCLDs are very variable between transplant centers [11,12], but it is estimated that they comprise at least 25% of LDKT programs [11]. Considering the donor perspective, two landmark studies from the last decade have shown an increased risk of ESRD in kidney donors compared with matched healthy non-donors [13,14]. These risks could be higher in MCLDs. The absence of high-quality studies with long-term follow-ups for recipient and donor outcomes makes this issue highly relevant to the transplant community [9,15].

A Kidney Disease: Improving Global Outcomes (KDIGO) work group published an extensive clinical practice guideline for evaluating LD candidates in 2017 [16]. A comprehensive approach to risk assessment is recommended in order to replace decisions based on the evaluation of isolated single risk factors, but precise orientations concerning MCLDs are lacking [16]. Data on organ admissibility for the intended recipients are even scarcer. Most of the available studies are retrospective, based on observational registry data or small cohort samples, or have considerable heterogeneity in design and results.

We sought to evaluate the effects on the graft outcomes (overall and censored graft failure (GF) and graft function) of clinical characteristics of LDs of medical complexity in our cohort, namely, donor age, reduced pre-donation eGFR, hypertension, dyslipidemia, smoking, proteinuria, and obesity. We hypothesize that these characteristics could be associated with worse outcomes.

## 2. Materials and Methods

We retrospectively reviewed the clinical data of all LDKT pairs that were performed at our center between January 1998 and January 2020 (*n* = 365). Inclusion criteria included at least one month of follow-up after KT. Thirteen patients were excluded from the analysis, eleven because of primary non-function due to perioperative problems, and two due to deaths within 30 days of the transplant (in all situations, donor-related characteristics causing the events were excluded). The remaining 352 pairs defined our study cohort.

The Institutional Review Board at our center, Centro Hospitalar Universitario de Santo António (CHUdSA), approved this retrospective observational study (Ref.: 147-21(119-DEFI/122-CE)), which was conducted according to the Helsinki declaration.

### 2.1. Donor Variables

Following international guidelines, all donors were subjected to a standard evaluation protocol [16,17]. Baseline demographic, anthropomorphic, analytical, and clinical data were collected from the LD. Serum creatinine-based CKD-EPI equation [18] was used to predict eGFR. Renal function was further evaluated by creatinine clearance in 24 h urine samples and serum C-cystatin in more recent donors. The final approval for kidney donation was reviewed in a multidisciplinary meeting, and ethical approval was mandatory.

In this study, we decided to use 90 mL/min/1.73 m^2^ as the eGFR cutoff, as the KDIGO guidelines propose [16]. In terms of donor age, the optimal cut-off point for the prediction of the outcomes of interest was set at 50 years through a univariate, time-dependent ROC analysis performed in this cohort (stroccurve STATA command).

Hypertension was defined by blood pressure (BP) in the consultation > 140/90 mmHg, 24 h ambulatory BP > 135/85 mmHg, and past hypertension diagnosis or antihypertensive medication [16]. Dyslipidemia was defined by the laboratory criteria (had total cholesterol > 200 mg/ dL, LDL > 130 mg/dL, triglycerides > 150 mg/dL, or HDL < 40 mg/dL) and/or the use of hypolipidemic agents. Upon urinary analysis, proteinuria was defined by random urine protein 0.15 to 0.5 g/g [16], and was confirmed by determination using a 24 h sample. Donors with confirmed proteinuria over 300 mg/day were discarded.

Left-side procurement was preferred for anatomical reasons, unless complex vessel anatomy or significant renal asymmetry were present. A transperitoneal laparoscopic approach was utilized in most donors. Lifetime annual follow-up appointments were available for all donors.

### 2.2. Recipient Variables

Demographic, analytical, and clinical data were collected from all the recipients. Comorbidities were registered. Chronic kidney disease (CKD) etiologies were grouped into six categories: diabetic nephropathy (DN), chronic glomerulonephritis (CGN), cystic diseases, urologic pathologies, unknown etiologies, and other causes. A serum creatinine based on the CKD-EPI equation [18] was used to predict the eGFR. Graft biopsies were performed for cause. Acute rejection (AR) was defined as per biopsy criteria. The recipients were followed until death, GF, or reaching 15 years of follow-up. Graft survival (GS) comprised the time from transplantation to GF, defined in the case of overall GS as a return to dialysis, retransplantation, or death with a functioning graft. Alternatively, in death-censored GS, GS was censored at the time of death with a functioning graft, but not imputed as a failure.

### 2.3. Transplant Variables

Two different transplant eras were considered in our cohort: 1998–2009 and 2010–2019. The peak-percentage-calculated panel-reactive antibody (cPRA) and the number of human leukocyte antigen (HLA) A, B, and DR mismatches were collected. Induction therapy was used in most patients with an anti-IL-2 receptor monoclonal antibody or a polyclonal anti-thymocyte globulin (ATG). ATG was primarily used in HLA-incompatible KT, sensitized patients, and retransplants. All enrolled recipients underwent similar triple-maintenance immunosuppression therapy consisting of a calcineurin inhibitor, mycophenolate mofetil, and prednisolone.

The incompatible group KT included cases of HLA-incompatible and ABO-incompatible KT. Both situations were submitted to desensitization protocols that included treatment with rituximab, plasmaphereses, and intravenous immunoglobulin. Further details of our immunosuppression protocols have been described previously [19].

### 2.4. Statistical Analysis

Continuous data were described using the mean and standard deviation (SD) or the median (interquartile range [IQR]), and categorical data were expressed as numbers (and percentages) as appropriate. Categorical data, including demographic, clinical, and immunological features, were compared using Pearson χ^2^ or Fisher’s exact tests. Continuous variables were compared using the Student *t* test or Mann—Whitney *U*, as appropriate. A comparison of annual graft function according to donor eGFR and age was performed by means of one-way ANOVA.

Overall and censored GS curves were visualized using the Kaplan–Meier method, with comparisons between the patients’ groups being carried out by log-rank tests. As our main purpose was to identify the clinical characteristics of LD associated with worse graft outcomes, all of these potential predictors were explored by multivariable Cox proportional hazards models adjusted to recipient and transplant variables and selected by a separate multivariable Cox model, in which a *p* value less than 0.157 was necessary for retention using a backward elimination method, as previously proposed [20]. Additionally, a double interaction term was included in the univariate and multivariable Cox models to examine the potential for effect modification between donor age and eGFR categories, given that they were identified as the main independent predictors of the outcomes of interest. Hazard proportionality was checked by plotting a log minus log of the distribution hazard and using Schoenfeld residuals of distribution hazards.

Statistical calculations were performed using STATA/MP, version 15.1 (Stata Corp, College Station, TX, USA). A 2-sided *p*-value < 0.05 was considered statistically significant.

## 3. Results

### 3.1. Baseline Characteristics

The baseline characteristics of our study cohort are summarized in Table 1. It was divided into two populations according to LD eGFR pre-donation: <90 vs. ≥90 mL/min/1.73 m^2^. Most donors (77%) had eGFR ≥ 90 mL/min/1.73 m^2^. Most recipients were male (67%), with a mean age of 40.4 ± 13.6 years old. The recipients from LD with higher eGFR (≥90 mL/min/1.73 m^2^) were significantly younger (39.6 ± 13.1 vs. 43.1 ± 14.6, *p* = 0.04) and less frequently had histories of coronary artery disease (6 vs. 13%, *p* = 0.017). Their donors were significantly younger (44.9 ± 10.4 vs. 52.8 ± 9.5, *p* < 0.001) and had fewer diagnoses of hypertension (13 vs. 23%, *p* = 0.019) and dyslipidemia (9 vs. 26%, *p* < 0.001).

The population was further stratified by donor age (< vs. ≥50 years) and eGFR pre-donation (<90 vs. ≥90 mL/min/1.73 m^2^), as depicted in Table 2. The population of recipients of younger donors (<50 years) with lower eGFRs presented significantly more frequent history of coronary artery disease vs. those with higher eGFRs (17 vs. 4%, *p* = 0.014). Remarkably, these donors were older (42.8 ± 5.4 vs. 39.1 ± 7.3, *p* = 0.011) and had significantly more frequent pre-donation diagnoses of hypertension (21 vs. 6%, *p* = 0.004).

#### 3.1.1. Graft Survival by Donor eGFR Pre-Donation

**Figure 1:**
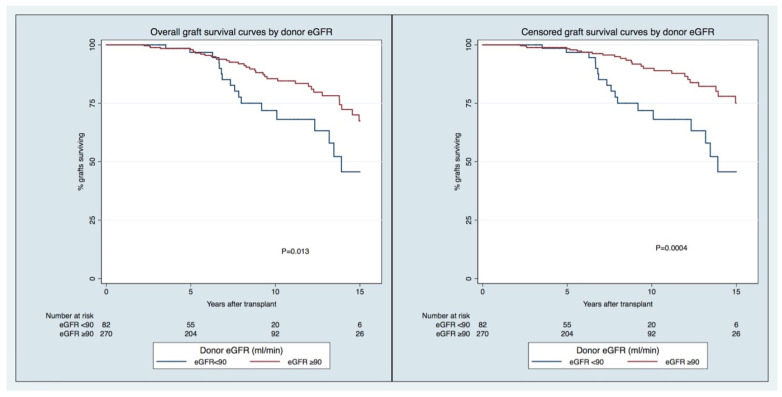
Overall (**right**) and censored (**left**) graft survival curves by donor eGFR pre-donation.

The overall GS rates in the group of recipients of transplants from LDs with higher vs. lower eGFRs (≥ vs. <90 mL/min/1.73 m^2^) at 5, 10, and 15 years were, respectively, 98/97%, 86/72%, and 67/46% (*p* = 0.013). The censored GS rates in the group of recipients of transplants from LDs with higher vs. lower eGFR (≥ vs. <90 mL/min/1.73 m^2^) at 5, 10, and 15 years were, respectively, 98/97%, 90/72%, and 75/46% (*p* = 0.0004). For both outcomes, the in-between groups’ differences were observed at a later stage (>5 years) of the follow-up (Figure 1).

#### 3.1.2. Independent Predictors of Graft Failure

Independent predictors of overall and censored GF, according to the recipient and KT variables, are shown in Table 3 and Table 4, respectively. Acute rejection and retransplant were significant predictors of both overall and censored GF. In contrast, recipient age, mismatch in DR, and CGN as the cause of CKD were only significant for censored GF.

Only donor age ≥ 50 years and pre-donation eGFR < 90 mL/min/1.73 m^2^ were identified as donor-derived independent predictors of overall or censored GF in the multivariable Cox models (Table 5 and Table 6). Remarkably, none of the remaining donor factors which were evaluated (hypertension, dyslipidemia, smoking, proteinuria, and obesity) were independent predictors for the outcomes of interest.

#### 3.1.3. Interaction of Pre-Donation eGFR × Donor Age

The interaction of LDs’ pre-donation eGFRs and ages was studied for both models (Table 7 and Table 8).

For the overall GF model, recipients of transplants from LDs < 50 years of age and those with eGFRs < 90 mL/min/1.73 m^2^ had the highest risk of overall GF, with an adjusted HR [95% CI] of 2.578 [1.120–5.795], compared to recipients of transplants from LDs < 50 years old and those with eGFRs ≥ 90 mL/min/1.73 m^2^. For the recipients of transplants from older donors (age ≥ 50), no difference in the risk of GF was observed between the eGFR categories.

Similarly, in the death-censored GF model, in younger donors (age < 50), recipients of transplants from LDs with eGFRs < 90 mL/min/1.73 m^2^ experienced the greatest risk of censored GF, with an adjusted HR [95%CI] of 3.216 [1.300–7.959], compared to recipients of transplants from LDs with eGFRs ≥ 90 mL/min/1.73 m^2^. In contrast, when the donors were older (age ≥ 50), no difference in the risk of GF was observed between the eGFR categories.

The interaction held when, in the univariate Cox model, the patients with acute rejection were excluded (Supplemental Materials). The multivariate models were adjusted for acute rejection (Table 7 and Table 8).

#### 3.1.4. The Longitudinal Pattern of Graft Function by LD eGFR and Age

**Figure 2:**
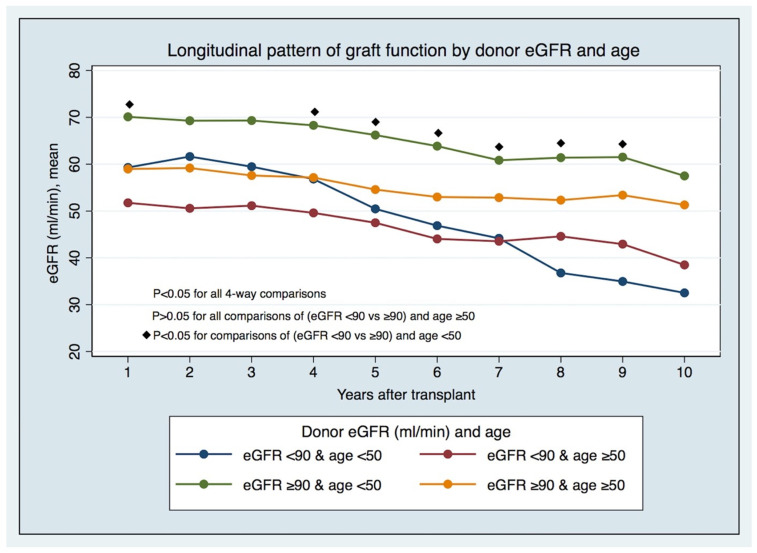
Longitudinal pattern of graft function by donor eGFR and age.

**Figure 3:**
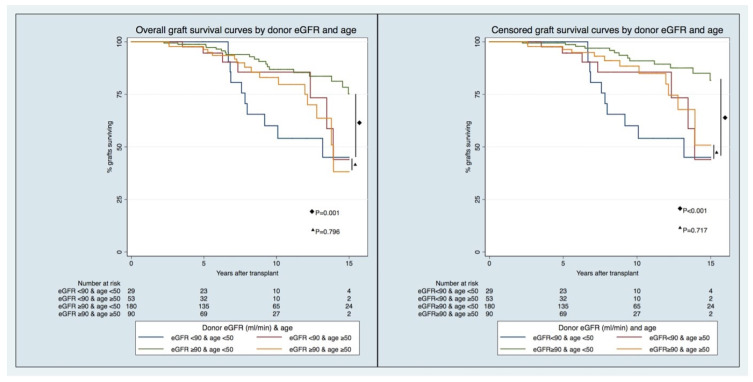
Overall (**right**) and censored (**left**) graft survival curves by donor eGFR and age.

The longitudinal pattern of graft function by LD eGFR pre-donation and age (Figure 2 and Supplemental Table S4) has displayed, since the first years after KT in recipients from younger LDs (age < 50), that significant differences exist between those with lower and higher eGFRs (eGFR < 90 vs. ≥90 mL/min/1.73 m^2^) pre-donation. In comparison, these differences do not hold in recipients from older LDs. The differences in prognoses in the recipients of transplants from LDs < 50 years of age with different eGFR categories are foreseeable early post-KT, and not just after five years, when there were different GS rates between groups (Figure 3).

## 4. Discussion

In this cohort of LDKT recipients, recipients from LDs with higher pre-donation eGFRs (≥90 mL/min/1.73 m^2^) had higher overall and censored GS rates compared to the recipients of those LDs with lower eGFRs (<90 mL/min/1.73 m^2^). Nevertheless, when the interaction between LD age and pre-donation eGFR was studied, recipients of transplants from younger LDs (<50) with lower eGFRs pre-donation experienced the highest risk of overall (adjusted HR [95% CI] 2.578 [1.120–5.795]) and censored GF (adjusted HR [95% CI] 3.216 [1.300–7.959]) compared to those with higher eGFRs pre-donation. Otherwise, when donors were older (≥50), no difference in the risk of GF was observed between eGFR categories. By further evaluating the longitudinal pattern of eGFR in the recipients, these differences between groups became evident from the first year after KT. No other analyzed factors (hypertension, obesity, proteinuria, or smoking habits) significantly influenced recipient outcomes in this cohort in either the univariate or multivariate analyses.

Lim et al. [21], in an analysis of 11,095 recipients of deceased donors and LDs, described an increased risk of overall GF in recipients with eGFRs < 30 mL/min/1.73 m^2^ vs. >60 mL/min/1.73 m^2^ at 12 months after KT, and the magnitude of the increased risk was higher among recipients from younger donors. In contrast, the donor type did not affect the risk, suggesting a possible donor-intrinsic pathology [21]. Our study showed a different relationship between LDs’ pre-donation eGFRs and graft outcomes modulated by LD age. The effect of pre-donation eGFR (< vs. ≥90 mL/min/1.73 m^2^) was different according to the LD subgroup age (<50 vs. ≥50), and this association has not been described yet. Our results suggest that the range of eGFR thresholds pursued for LD should be adjusted to their age.

The age cutoff for “old” LDs remains unsolved. If the Japanese Transplantation Committee were to define standard LDs as those aged up to 70 years [22], other guidelines would use thresholds such as 45 [23], 50 [24], 55 [25], and 60 years [26]. We used 50 years as an exploratory cutoff based on the statistical approach mentioned above. The reported outcomes also differ. Some studies have described inferior graft and patient survival [27], death-censored graft survival [28], and higher AR [27], or no significant difference [29,30], among the recipients of kidneys from “older” LDs. In their metanalysis, Bellini et al. refer to high heterogeneity between studies [9].

Our results are different from previous publications. Although, as expected, our donors’ ages impacted our recipients’ outcomes, when an interaction with pre-donation eGFR was studied, a dissimilarity in outcomes driven by pre-donation renal function between younger and older LDs became evident. This interaction held true when the impact of AR was further analyzed in a univariate Cox model excluding patients who experienced AR. AR was a variable that was accounted for in the multivariate Cox model. This may result from different pathophysiologic statuses being measured as pre-donation eGFR.

The evaluation of renal function is critical in LD evaluation. It is used to screen for kidney disease and aid in predicting graft function and long-term kidney failure risk after donation. The measurement of GFR using an “ideal” filtration marker is unfeasible in clinical practice; determining eGFR from serum levels of endogenous filtration markers, such as creatinine [16], is the most widely used approach, and it is recommended by the current guidelines [16,17]. The KDIGO guidelines consider an eGFR of ≥90 mL/min/1.73 m^2^ acceptable for donation, and an eGFR60 < mL/min/1.73 m^2^ is considered a contraindication for donation [16]. Our results remind us to focus on younger LDs, in whom we should be alert to lower, yet almost normal, pre-donation eGFRs, as these could translate into unrecognized pathologies.

Based on the KDIGO guidelines [31], the current definition of CKD includes the diminution of GFR below 60 mL/min/1.73 m^2^. This threshold does not consider the physiologic decline of GRF with healthy aging. The available evidence suggests that the GFR threshold below which the risk of mortality is increased is different across ages, from 75 mL/min/1.73 m^2^ in younger persons to values as low as 45 mL/min/1.73 m^2^ in elderly people [32]. Structural differences are identified between aging kidneys and CKD. In aging kidneys, substantial nephron loss is expected, albeit with no compensation by the remaining nephrons. In CKD, beyond disease-specific pathology, it is expected that associated glomerular enlargement, segmental glomerulosclerosis, and higher single-nephron GFR in intact nonsclerotic glomeruli will occur as adaptative changes [32]. The fixed GFR threshold may result in the overdiagnosis of CKD in older adults, but it may also lead to missed diagnoses of CKD in younger individuals without obvious signs of kidney damage and who have a GFR well above that threshold. This group may include young people with low nephron reserves, those born pre-term, or those with other unrecognized risk factors who may have a risk of developing CKD and associated comorbidities [32]. Some of our younger LDs with lower eGFR could be part of this group.

Some transplant centers perform routine implantation biopsies, Structural findings in the kidney could provide insight into the pathology of early CKD and help us to understand our findings. Still, this practice is not universal; it has some risks, and the biopsies are performed after donor selection. A study by Elsherbiny et al. [33] evaluated 1395 LDKT implantation biopsies of donated kidneys. It concluded that kidney function and CKD risk factors had similar associations with different morphologic measures of nephron hypertrophy, but different associations with glomerulosclerosis [33]. The authors suggested that different biologic pathways may contribute to the risk of CKD [33]. Exploring biomarkers and metabolic pathways beyond serum creatinine and eGFR that could help us to understand and predict the strength of these correlations before donation, thus helping in terms of accepting or discarding some donors, are currently major challenges of LDKT programs.

The available evidence regarding the outcomes of grafts with lower baseline function is scarce. Norden et al. [34] reported that a donor with low GFR was associated with a higher graft loss risk, establishing a lower acceptance limit of 80 mL/min in many transplant centers. Young et al. [35], in an analysis of 2057 LDKT pairs, reported no differences in the adjusted hazard ratios for GF amongst recipients of LD kidneys with eGFRs ≥ 110 mL/min/1.73 m^2^ vs. <80 mL/min/1.73 m^2^. In our cohort, GS was significantly higher in the group of recipients of LD with higher pre-donation eGFRs (eGFR ≥ 90 mL/min/1.73 m^2^) after the first five years. Nevertheless, when the interaction between LD age and pre-donation eGFR was studied, this held only for recipients of transplants from younger LDs (<50 years).

We did not find a significant effect on graft outcomes for the other factors studied (hypertension, dyslipidemia, smoking, proteinuria, and obesity), or for the association of factors, although their impact has been described in other cohorts [9,23,36]. Remarkably, in the “problem” group (age < 50 and eGFR < 90 mL/min/1.73 m^2^), LDs had significantly more diagnoses of hypertension (21 vs. 6%, *p* = 0.004) vs. those in the same age group = with higher eGFRs, which could also indicate unrecognized kidney disease. Their recipients had significantly more frequent coronary artery disease, but they had more GF, not more deaths. Hypertension is the strongest cardiovascular risk factor worldwide, and is also significantly associated with CKD [37]. Conversely, there are limited data on the association between LD hypertension and recipient outcomes. Both a significant impact [23] and no impact [38] in the recipients of hypertensive LD kidneys after KT have been described. Hypertensive donors with well-controlled hypertension, without evidence of end organ damage or metabolic syndrome, were accepted after a comprehensive evaluation and informed consent in our cohort, according to published guidelines [16,17]. We did not find significant effects of LD hypertension on graft outcomes as other studies did [38]. We can hypothesize that LDs are otherwise healthy people without evidence of end-organ damage, and only selected hypertensive subjects were effective donors.

Obesity is a worldwide pandemic. It is associated with structural, hemodynamic, and metabolic alterations in the kidneys. Despite this, several studies, including ours, have failed to find significance in the immediate and short-term post-transplant recipient outcomes between normal-weight and overweight and obese donors [36]. We must note that we only accepted those obese donors with BMIs near 30 who showed the ability to lose weight and maintain a lower weight before donating. Other cardiovascular risk factors, such as concomitant dyslipidemia, smoking habits, and impaired glucose balance, can influence the decision to accept an LD.

Often, potential donors exhibit several medical complexities. Strong outcome data are not available. In clinical practice, we perform a global risk assessment considering the global risk for the donor, including the expected lifetime exposure to the risk and the adequacy of the graft for the recipient. Theoretically, the more medically complex a donor is, the more likely the donor or the corresponding recipient is to have an inferior outcome. We were unable to demonstrate this in our cohort analysis 

Finally, we should emphasize the implications of our study results. Recipients from LDs with lower eGFRs (<90 mL/min/1.73 m^2^) had worse outcomes, but this effect was modulated by the LDs’ ages, and was only significant in recipients from younger donors (<50 y). This effect was evident from the first year after KT onward in the evolution of kidney function. These results agree with the current guidelines for LD evaluation [16,17], which recommend a comprehensive approach to risk assessment in order to replace decisions based on assessments of single risk factors in isolation. Although these recommendations are centered on donor safety, the same strategy should be followed in the recipient pair selection process to optimize the results of the procedure.

Our study has several limitations. First, donors were evaluated retrospectively, and unobserved confounders may have introduced bias. Second, our cohort consisted only of Caucasian patients, limiting the generality of our results. In addition, using eGFR to assess kidney function by means of estimation equations has limitations, but it is the common practice in most transplant centers and agrees with the International Guidelines [16,17]. The defined cohorts were not wholly balanced considering the preoperative variables, as depicted in Table 1 and Table 2. A multivariable analysis was performed, considering transplant, recipient, and donor factors, to overcome this limitation. In addition, added strengths of our study cohort are its larger size and reasonable length of follow-up. Considering two time periods can partially offset some heterogeneity about immunosuppressive protocols during those periods. Nevertheless, longer follow-up studies must be required; prospective studies are necessary to allow for a cause–effect analysis of the studied parameters. We recognize that the most important issue in LDKT is assuring LDs’ safety, but this goes beyond the scope of this article and deserves a separate analysis. Additionally, we did not seek to compare the effects of declining a “complex” living donor and causing a patient to remain on the waiting list, but rather sought to better characterize that phenotype.

## 5. Conclusions

In our cohort, pre-donation eGFR was associated with post-transplant outcomes, but the donor’s age significantly interacted with this effect. In younger donors only, an eGFR < 90 vs. ≥90 mL/min/1.73 m^2^ had a detrimental impact on graft survival, drawing attention to the need for an age-adjusted eGFR cutoff for donor admissibility. The dissimilarity in outcomes driven by pre-donation renal function between younger and older donors may result from different pathophysiologic statuses being measured as eGFR pre-donation.

## Figures and Tables

**Table 1:** Baseline characteristics of the cohort.

	Total *n* = 352	eGFR < 90 mL/min/1.73 m^2^ *n* = 82 (23%)	eGFR ≥ 90 mL/min/1.73 m^2^ *n* = 270 (77%)	*p*
Recipient				
Age, mean ± SD	40.4 ± 13.6	43.1 ± 14.6	39.6 ± 13.1	**0.040**
F sex, *n* (%)	117 (33)	24 (29)	93 (34)	0.384
BMI, mean ± SD	23.6 ± 3.7	23.4 ± 3.6	23.6 ± 3.8	0.581
DM, *n* (%)	20 (6)	7 (9)	13 (5)	0.202
Smoking habits, *n* (%)	80 (23)	17 (21)	63 (23)	0.622
Coronary heart disease, *n* (%)	26 (7)	11 (13)	15 (6)	**0.017**
Cerebrovascular disease *n* (%)	5 (1)	2 (2)	3 (1)	0.331
CKD etiology, *n* (%)				0.614
DN	10 (3)	3 (4)	7 (3)	
CGN	166 (47)	40 (49)	126 (47)	
Hereditary	16 (5)	4 (5)	12 (4)	
Cystic disease	36 (10)	8 (10)	28 (10)	
Urologic	57 (16)	13 (16)	44 (16)	
Unknown	57 (16)	10 (12)	47 (17)	
Others	10 (3)	4 (5)	6 (2)	
Vintage (years), median (IQR)	1.17 (0.28–2.59)	1.19 (0.10–2.52)	1.17 (0.29–2.70)	0.656
Previous RRT, *n* (%)				0.642
HD	200 (57)	44 (54)	156 (58)	
PD	79 (22)	18 (22)	61 (23)	
Preemptive	73 (21)	20 (24)	53 (20)	
Donor				
Age, mean ± SD	46.8 ± 10.7	52.8 ± 9.5	44.9 ± 10.4	**<0.001**
Age ≥ 50, *n* (%)	143 (41)	53 (65)	90 (33)	**<0.001**
F sex *n* (%)	244 (69)	55 (67)	189 (70)	0.615
BMI, mean ± SD	25.2 ± 3.3	25.8 ± 3.1	25.0 ± 3.4	0.067
BMI ≥ 30, *n* (%)	33 (9)	10 (12)	23 (9)	0.317
Pre-donation SCr, median (IQR)	0.72 (0.64–0.88)	0.90 (0.80–1.02)	0.69 (0.61–0.78)	**<0.001**
Pre-donation eGFR, mean ± SD	100.5 ± 14.7	79.7 ± 8.4	106.8 ± 9.5	**<0.001**
Hypertension, *n* (%)	53 (15)	19 (23)	34 (13)	**0.019**
Dyslipidemia, *n* (%)	46 (13)	21 (26)	25 (9)	**<0.001**
Smoking habits *n* (%)	56 (16)	9 (11)	47 (17)	0.163
ProtU 0.15–0.5 g/g, *n* (%)	98 (28)	23 (28)	75 (28)	0.962
Transplant				
Transplant era, *n* (%)				0.624
1998–2009	102 (29)	22 (27)	80 (30)	
2010–2019	250 (71)	60 (73)	190 (70)	
LUR, *n* (%)	123 (35)	31 (38)	92 (34)	0.535
Previous transplant, *n* (%)	64 (18)	18 (22)	46 (17)	0.312
PRA-CDC, *n* (%)				0.695
0–4	314 (89)	74 (90)	240 (89)	
5–49	28 (8)	5 (6)	23 (9)	
50–100	10 (3)	3 (4)	7 (3)	
HLA-incompatible, *n (*%)	26 (7)	7 (9)	19 (7)	0.649
ABO-incompatible, *n* (%)	15 (4)	2 (2)	13 (5)	0.535
HLA AB MM, mean ± SD	2.03 ± 1.18	1.98 ± 1.18	2.04 ± 1.18	0.648
HLA DR MM, mean ± SD	0.97 ± 0.68	0.99 ± 0.69	0.96 ± 0.68	0.776
Induction, *n* (%)				0.699
No	16 (5)	3 (4)	13 (5)	
Basiliximab	273 (78)	62 (76)	211 (78)	
ATG	63 (18)	17 (21)	46 (17)	
Acute rejection, *n* (%)	47 (13)	12 (15)	35 (13)	0.697
Censored graft failure *n* (%)	42 (12)	17 (21)	25 (9)	**0.005**
Overall graft failure *n* (%)	52 (15)	17 (21)	35 (13)	0.082
Years of follow-up, median (IQR)	7.4 (4.9–11.5)	6.6 (4.5–9.5)	7.8 (5.1–12.0)	**0.041**

F—female; BMI—body mass index; DM—diabetes mellitus; CKD—chronic kidney disease; DN—diabetic nephropathy; CGN—chronic glomerulonephritis; SD—standard deviation; IQR—interquartile range; ProtU—proteinuria; LUR—living unrelated donor; PRA—panel-reactive antibody; CDC—complement-dependent cytotoxicity; ATG—anti-thymocyte thymoglobin; eGFR—estimated glomerular filtration rate; statistically significant values were represented on bold.

**Table 2:** Baseline characteristics according to eGFR and stratified by donor age.

	Donor Age < 50 *n* = 209			Donor Age ≥ 50 *n* = 143		
	eGFR < 90 mL/min/1.73 m^2^ *n* = 29 (14%)	eGFR ≥ 90 mL/min/1.73 m^2^ *n* = 180 (86%)	*p*	eGFR < 90 mL/min/1.73 m^2^ *n* = 53 (37%)	eGFR ≥ 90 mL/min/1.73 m^2^ *n* = 90 (63%)	*p*
Recipient						
Age, mean ± SD	38.0 ± 13.0	38.0 ± 12.2	0.985	46.0 ± 14.8	42.9 ± 14.3	0.234
F sex, *n* (%)	8 (28)	64 (36)	0.402	16 (30)	29 (32)	0.800
BMI, mean ± SD	23.2 ± 2.8	23.6 ± 3.8	0.525	23.6 ± 3.9	23.7 ± 3.8	0.914
DM, *n* (%)	1 (4)	9 (5)	1	6 (11)	4 (4)	0.173
Smoking habits, *n* (%)	2 (7)	41 (23)	0.051	15 (28)	22 (24)	0.611
Coronary heart disease, *n* (%)	5 (17)	7 (4)	**0.014**	6 (11)	8 (9)	0.636
Cerebrovascular disease, *n* (%)	1 (3)	3 (2)	0.452	1 (2)	0 (0)	0.371
CKD etiology, *n* (%)			0.865			0.475
DM	1 (3)	4 (2)		2 (4)	3 (3)	
CGN	15 (52)	90 (50)		25 (47)	36 (40)	
Hereditary	3 (10)	10 (6)		1 (2)	2 (2)	
Cystic disease	1 (3)	11 (6)		7 (13)	17 (19)	
Urologic	5 (17)	27 (15)		8 (15)	17 (19)	
Unknown	4 (14)	33 (18)		6 (11)	14 (16)	
Others	0 (0)	5 (3)		4 (8)	1 (1)	
Vintage (years), median (IQR)	1.36 (0.78–2.52)	1.11 (0.29–2.35)	0.213	1.14 (0–1.83)	1.40 (0.31–4.11)	0.082
Previous RRT, *n* (%)			0.642			0.737
HD	17 (59)	106 (59)		27 (51)	50 (56)	
PD	7 (24)	41 (23)		11 (21)	20 (22)	
Preemptive	5 (17)	33 (18)		15 (28)	20 (22)	
Donor						
Age, mean ± SD	42.8 ± 5.4	39.1 ± 7.3	**0.011**	58.4 ± 6.0	56.5 ± 4.2	**0.029**
F sex *n* (%)	18 (62)	119 (66)	0.671	37 (70)	70 (78)	0.289
BMI, mean ± SD	25.3 ± 2.9	24.8 ± 3.4	0.483	26.1 ± 3.1	25.5 ± 3.4	0.313
BMI ≥ 30, *n* (%)	3 (10)	15 (8)	0.721	7 (13)	8 (9)	0.416
Pre-donation SCr, median (IQR)	1.00 (0.88–1.06)	0.70 (0.63–0.80)	**<0.001**	0.88 (0.69–0.97)	0.66 (0.60–0.74)	**<0.001**
Pre-donation eGFR, mean ± SD	80.9 ± 7.2	110.3 ± 9.2	**<0.001**	79.1 ± 9.0	99.9 ± 5.6	**<0.001**
Hypertension, *n* (%)	6 (21)	10 (6)	**0.004**	13 (25)	24 (27)	0.778
Dyslipidemia, *n* (%)	3 (10)	10 (6)	0.397	18 (34)	15 (17)	**0.018**
Smoking habits, *n* (%)	3 (10)	38 (21)	0.215	6 (11)	9 (10)	0.803
ProtU 0.15–0.5 g/g, *n* (%)	9 (31)	53 (29)	0.862	14 (26)	22 (24)	0.793
Transplant						
Transplant era, *n* (%)			0.104			0.237
1998–2009	14 (48)	59 (33)		8 (15)	21 (23)	
2010–2019	15 (52)	121 (67)		45 (85)	69 (77)	
LUR, *n* (%)	8 (28)	57 (32)	0.660	23 (43)	35 (39)	0.596
Previous transplant, *n* (%)	6 (21)	25 (14)	0.339	12 (23)	21 (23)	0.924
PRA-CDC, *n* (%)			0.439			0.793
0–4	25 (86)	161 (89)		49 (92)	79 (88)	
5–49	2 (7)	14 (8)		3 (6)	9 (10)	
50–100	2 (7)	5 (3)		1 (2)	2 (2)	
HLA-incompatible, *n* (%)	2 (7)	12 (7)	1	5 (9)	7 (8)	0.761
ABO-incompatible, *n* (%)	2 (7)	10 (6)	0.675	0 (0)	3 (3)	0.295
HLA AB MM, mean ± SD	2.17 ± 0.97	2.06 ± 1.20	0.668	1.87 ± 1.27	2.02 ± 1.15	0.464
HLA DR MM, mean ± SD	1.03 ± 0.63	0.97 ± 0.69	0.641	0.96 ± 0.73	0.94 ± 0.68	0.898
Induction, *n* (%)			0.102			0.648
No	3 (10)	11 (6)		0 (0)	2 (2)	
Basiliximab	16 (55)	133 (74)		46 (87)	78 (87)	
ATG	10 (34)	36 (20)		7 (13)	10 (11)	
Acute rejection, *n* (%)	6 (21)	24 (13)	0.294	6 (11)	11 (12)	0.872
Censored graft failure *n* (%)	10 (34)	13 (7)	**<0.001**	7 (13)	12 (13)	0.983
Overall graft failure *n* (%)	10 (34)	19 (11)	**0.001**	7 (13)	16 (18)	0.472
Years of follow-up, median (IQR)	7.8 (5.3–12.1)	8.1 (4.9–12.5)	0.687	5.6 (4.0–8.5)	7.8 (5.4–10.8)	**0.008**

F—female; BMI—body mass index; DM—diabetes mellitus; CKD—chronic kidney disease; CGN—chronic glomerulonephritis; SD—standard deviation; IQR—interquartile range; ProtU—proteinuria; LUR—living unrelated donor; PRA—panel-reactive antibody; CDC—complement-dependent cytotoxicity; ATG—anti-thymocyte thymoglobin; eGFR—estimated glomerular filtration rate, statistically significant values were represented on bold.

**Table 3:** Predictors of overall graft failure included if *p* < 0.157 in a stepwise backward selection (variables from the recipient/kidney transplant).

	HR	95% CI	*p*
Acute rejection	3.115	1.834–5.991	<0.001
Female recipient	1.584	0.898–2.793	0.112
Retransplant	2.672	1.483–4.816	0.001

HR—hazard rate; CI—confidence interval.

**Table 4:** Predictors of censored graft failure included if *p* < 0.157 in a stepwise backward selection (variables from the recipient/kidney transplant).

	HR	95% CI	*p*
Acute rejection	2.095	1.017–4.314	0.045
Retransplant	3.385	1.718–6.670	<0.001
Recipient age	0.943	0.911–0.976	0.001
MM DR	1.631	0.913–2.912	0.098
CGN etiology	2.041	1.004–4.147	0.049

HR—hazard rate; CI—confidence interval; MM—mismatch; CGN—chronic glomerulonephritis.

**Table 5:** Predictors of overall graft failure; multivariable Cox model.

	HR	95% CI	*p*
Pre-donation eGFR < 90 mL/min/1.73 m^2^	1.893	1.030–3.478	0.040
Donor age ≥ 50 years	1.730	0.947–3.159	0.075
Hypertension	0.523	0.200–1.370	0.187
Dyslipidemia	0.653	0.233–1.825	0.416
Smoking habits	1.159	0.452–2.971	0.759
Donor BMI ≥ 30 kg/m^2^	2.087	0.774–5.626	0.146
Proteinuria 0.15–0.5 g/24 h	1.551	0.846–2.843	0.156
Female donor	1.227	0.594–2.538	0.581
Acute rejection	3.316	1.826–6.020	<0.001
Female recipient	1.662	0.935–2.956	0.084
Retransplant	2.643	1.453–4.810	0.001

HR—hazard rate; CI—confidence interval; eGFR—estimated glomerular filtration rate; BMI—body mass index.

**Table 6:** Predictors of censored graft failure; multivariable Cox model.

	HR	95% CI	*p*
Pre-donation eGFR < 90 mL/min 1.73 m^2^	2.378	1.224–4.622	0.011
Donor age ≥ 50 years	2.108	1.0.43–4.260	0.038
Hypertension	1.081	0.385–3.034	0.883
Dyslipidemia	0.658	0.218–1.985	0.457
Smoking habits	2.093	0.682–6.424	0.197
Donor BMI ≥ 30 kg/m^2^	2.103	0.750–5.894	0.158
Proteinuria 0.15–0.5 g/24 h	1.250	0.595–2.626	0.556
Female donor	1.400	0.597–3.288	0.439
Acute rejection	2.137	1.109–4.479	0.044
Retransplant	3.458	1.715–6.975	0.001
Recipient age	0.945	0.911–0.980	0.002
MM DR	1.538	0.847–2.795	0.158
CGN etiology	2.529	1.202–5.323	0.015

HR—hazard rate; CI—confidence interval; eGFR—estimated glomerular filtration rate; BMI: body mass index; MM DR: mismatch in DR; CGN—chronic glomerulonephritis.

**Table 7:** Interaction between donor age and eGFR categories for the prediction of overall graft failure: multivariate Cox model (adjusted to the same variables as Table 5).

Multivariate Cox Model P Interaction = 0.317	HR	95% CI	*p*
eGFR < 90 vs. ≥90 and donor age < 50 years	2.578	1.120–5.795	0.026
GFR < 90 vs. ≥90 and donor age ≥ 50 years	1.328	0.548–3.405	0.555

HR—hazard rate; CI—confidence interval eGFR—estimated glomerular filtration rate. Units: mL/min/1.73 m^2^.

**Table 8:** Interaction between donor age and eGFR categories for the prediction of censored graft failure: multivariate Cox model (adjusted to the same variables as Table 6).

Multivariate Cox Model P Interaction = 0.349	HR	95% CI	*p*
eGFR < 90 vs. ≥90 and donor age < 50 years	3.216	1.300–7.959	0.012
GFR < 90 vs. ≥90 and donor age ≥ 50 years	1.667	0.607–4.582	0.322

eGFR—estimated glomerular filtration Rate. Units: mL/min/1.73 m^2^.

## Data Availability

The data underlying this article will be shared upon reasonable request to the corresponding author.

## Supplementary Materials

The following supporting information can be downloaded at: https://www.mdpi.com/article/10.3390/jcm12216777/s1, Table S1. Overall and censored graft and survival by pre-donation LD eGFR and Age, Table S2. Interaction between donor age and eGFR categories for the prediction of overall graft failure, Table S3. Interaction between donor age and eGFR categories for the prediction of censored graft failure, Table S4. Longitudinal pattern of graft function by graft eGFR and age.
