# Supplementary material for: Living Donors’ Age Modifies the Impact of Pre-Donation Estimated Glomerular Filtration Rate on Graft Survival
Source: J Clin Med. 2023 Oct 26;12(21):6777. doi: 10.3390/jcm12216777 (PMC10649187; doi:10.3390/jcm12216777)
Supplement: Supplementary file 1 [file jcm-12-06777-s001.zip › jcm-2637306-supplementary.pdf]

Table S1. Overall and censored graft and survival by pre-donation LD eGFR and Age

	5y				10y				15y			
**eGFR**	**<90 ***		**≥90***		**<90***		**≥90***		**<90 ***		**≥90***	
**Age**	**<50**	**≥50**	**<50**	**≥50**	**<50**	**≥50**	**<50**	**≥50**	**<50**	**≥50**	**<50**	**≥50**
Overall graft survival	100%	95%	99%	96%	60%	86%	87%	83%	45%	44%	75%	38%
Censored graft survival	100%	95%	99%	96%	60%	87%	91%	88%	45%	44%	82%	51%

eGFR estimated Glmerular filtration rate; Years after kidney transplant; **ml/min/1.73m^2*

Table S2 Interaction between donor age and eGFR categories for the prediction of overall graft failure.

Table S2.1. Univariate Cox Model

Univariate Cox Model<br>*P interaction=0.041*	HR	95% CI	P
eGFR<90 vs. ≥90 & Donor Age<50 years	3.366	1.564-7.246	**0.002**
eGFR<90 vs. ≥90 & Donor Age ≥50 Years	0.984	0.404-2.401	0.972

eGFR estimated Glomerular Filtration Rate; Units: ml/min/1.73m^2.

Table S2.2. Univariate Cox Model (excluding recipients who experienced acute rejection, n=47)

Univariate Cox Model<br>*P interaction=0.150*	HR	95% CI	P
eGFR<90 vs. ≥90 & Donor Age<50 years	3.150	1.107-8.961	**0.002**
GFR<90 vs. ≥90 & Donor Age ≥50 Years	1.041	0.353-3.067	0.942

eGFR estimated Glomerular Filtration Rate; Units: ml/min/1.73m^2.

Table S3 Interaction between donor age and eGFR categories for the prediction of censored graft failure.

Table S3.1*.* Univariate Cox Model

Univariate Cox Model<br><br>*P interaction=0.039*	HR	95% CI	P
eGFR<90 vs. ≥90 & Donor Age<50 years	4.923	2.156-11.239	**<0.001**
GFR<90 vs. ≥90 & Donor Age ≥50 Years	1.316	0.516-3.355	0.566

eGFR estimated Glomerular Filtration Rate; Units: ml/min/1.73m^2.

Table S3.2*. Univariate Cox Model (excluding recipients who experienced acute rejection, n=47)*

Univariate Cox Model<br><br>*P interaction=0.196*	HR	95% CI	P
eGFR<90 vs. ≥90 & Donor Age<50 years	4.207	1.406-12.584	**0.010**
GFR<90 vs. ≥90 & Donor Age ≥50 Years	1.465	0.461-4.657	0.518

eGFR estimated Glomerular Filtration Rate; Units: ml/min/1.73m^2.

Table S4 Longitudinal pattern of graft function by graft eGFR and age

	N	Total Mean±SD	1.eGFR <90 & age <50, Mean±SD	2.eGFR <90 & age ≥50 Mean±SD	3.eGFR >90 & age <50 Mean±SD	4.eGFR ≥90 & age ≥50 Mean±SD	P overall	P 1 vs 3	P 2 vs 4
Year 1	347	64±19	59±18	52±15	70±20	59±16	<0.001	0.018	0.135
Year 2	325	63±19	62±19	51±16	69±19	59±16	<0.001	0.251	0.052
Year 3	312	63±20	59±18	51±15	69±21	58±17	<0.001	0.074	0.400
Year 4	280	62±20	57±21	50±17	68±20	57±19	<0.001	0.040	0.287
Year 5	252	59±21	50±21	46±17	66±21	55±19	<0.001	0.006	0.293
Year 6	218	57±22	47±24	42±18	64±20	53±20	<0.001	0.004	0.197
Year 7	210	55±22	44±26	42±19	61±21	53±21	<0.001	0.009	0.206
Year 8	204	54±23	37±26	42±20	61±22	52±21	<0.001	<0.001	0.414
Year 9	195	54±24	35±30	40±19	62±23	53±21	<0.001	<0.001	0.158
Year 10	121	51±36	33±30	38±22	57±26	51±55	0.044	0.061	1.000

eGFR estimated Gomerular Filtration Rate;Units: age- years old; *eGFR: ml/min/1.73m^2*
